# Structured light for touchless 3D registration in video-based surgical navigation

**DOI:** 10.1007/s11548-024-03180-5

**Published:** 2024-05-30

**Authors:** Tânia Baptista, Miguel Marques, Carolina Raposo, Luís Ribeiro, Michel Antunes, Joao P. Barreto

**Affiliations:** 1 Institute of Systems and Robotics, University of Coimbra, Coimbra, Portugal; 2 Perceive3D, Coimbra, Portugal

**Keywords:** Structured light, Surgical navigation, Deep learning, Arthroscopy

## Abstract

**Purpose:**

Arthroscopic surgery, with its inherent difficulties on visibility and maneuverability inside the joint, poses significant challenges to surgeons. Video-based surgical navigation (VBSN) has proven to have clinical benefits in arthroscopy but relies on a time-consuming and challenging surface digitization using a touch probe to accomplish registration of intraoperative data with preoperative anatomical models. This paper presents an off-the-shelf laser scanner for noninvasive registration that enables an increased area of reachable region.

**Methods:**

Our solution uses a standard arthroscope and a light projector with visual markers for real-time extrinsic calibration. Nevertheless, the shift from a touch probe to a laser scanner introduces a new challenge—the presence of a significant amount of outliers resulting from the reconstruction of nonrigid structures. To address this issue, we propose to identify the structures of interest prior to reconstruction using a deep learning-based semantic segmentation technique.

**Results:**

Experimental validation using knee and hip phantoms, as well as ex-vivo data, assesses the laser scanner’s effectiveness. The integration of the segmentation model improves results in ex-vivo experiments by mitigating outliers. Specifically, the laser scanner with the segmentation model achieves registration errors below 2.2 mm, with the intercondylar region exhibiting errors below 1 mm. In experiments with phantoms, the errors are always below 1 mm.

**Conclusion:**

The results show the viability of integrating the laser scanner with VBSN as a noninvasive and potential alternative to traditional methods by overcoming surface digitization challenges and expanding the reachable region. Future efforts aim to improve hardware to further optimize performance and applicability in complex procedures.

**Supplementary Information:**

The online version contains supplementary material available at 10.1007/s11548-024-03180-5.

## Introduction

**Fig. 1:**
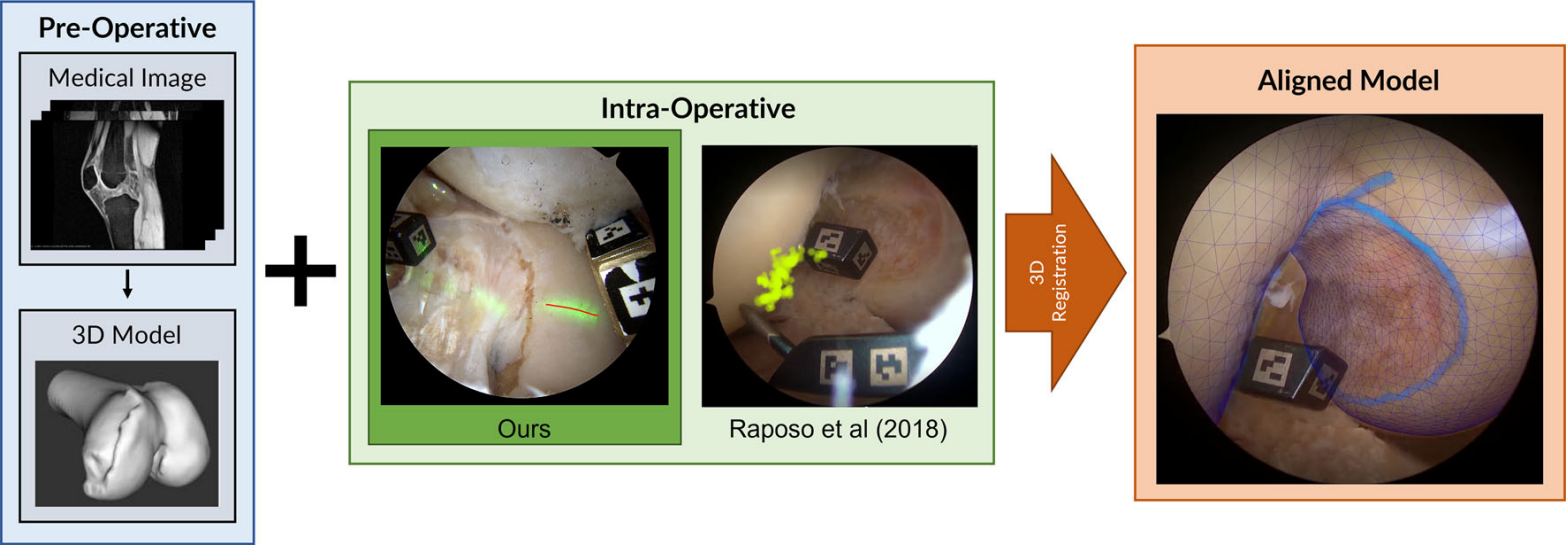
The existing solution for VBSN uses information from preoperative imaging, such as MRI, and requires the surgeon to digitize the surface of the bone with a touch probe, enabling the 3D reconstruction of points on bone surface [16]. This paper proposes to replace the use of a touch probe by an off-the-shelf laser scanner to reconstruct the points. After 3D registration is completed, the preoperative model can be overlaid with the anatomy using augmented reality. For better assessment of the registration quality, the preoperative model (shown as a mesh grid on the right) is augmented with a blue contour representing the intercondylar arc

Contrary to optical tracking [8], video-based surgical navigation (VBSN) [16] leverages visual markers attached to the patient’s anatomy to guide the surgeon throughout the medical procedure. When applied to arthroscopic procedures, these markers are placed inside the joint, precluding the need for additional external incisions. The video-based navigation process entails the precise registration of a preoperative anatomical model with data acquired intraoperatively (Fig. 1). The registration process requires the surgeon to digitize the surface of interest that corresponds to the preoperative model. Due to challenging conditions of arthroscopic scenarios, namely the limited manoeuverability and visibility inside the joint, the existence of floating particles and tissue, the illumination changes, and the distortion induced by arthroscopic lenses, acquiring sufficient points on the bone surface for accurate registration can be a very time-consuming and error-prone process.

In contrast to arthroscopic procedures involving the knee joint, where the bone and cartilage surfaces are readily accessible, femoral acetabular impingement (FAI) surgeries [1] present unique challenges. The bone and cartilage areas in FAI are notably more difficult to access, and their spherical geometry causes ambiguities that further complicate the registration process. In addition to these difficulties, there is also the concern of medical practitioners regarding the digitization of the cartilage surface. This process can potentially damage the cartilage, leading to patient discomfort and accelerate the onset of osteoarthritis.

This paper introduces a novel system that addresses the previously mentioned challenges. Rather than relying on the digitizing of the articular surface using a touch probe for generating intraoperative 3D point data, we propose the use of a simple and affordable laser scanner (Fig. 1). An off-the-shelf laser projector is used to project a contour onto the anatomical surface, which is subsequently detected in the arthroscopic video using an image processing technique. By tracking the visual markers attached to the laser projector at each frame-time instant, it is possible to determine the 3D position of the identified laser contour in the reference frame of the camera. By simultaneously tracking the visual marker that is attached to the anatomy, the reconstructed contour can be represented in the reference frame of the patient and subsequently registered with the preoperative model. This new approach for arthroscopic navigation, referred to as touchless registration, offers several advantages. Touchless registration is more efficient, eliminating the time-consuming process of physical digitization. Moreover, it eliminates any risk of bone or cartilage damage since there is no physical contact. Since there is no need for physical interaction with a specialized contact tool, it increases the accessible area for the surgeon. By providing a larger area for reconstruction, touchless registration opens the way for FAI surgery and other procedures, where it is difficult to access the target anatomy.

While touchless registration effectively addresses the issues discussed previously, it introduces a new challenge. Unlike manual digitization where the surgeon only focuses on the target surfaces contained in the preoperative model, the proposed structured light (SL) system projects a contour independently of the surface of interest. Consequently, this approach will reconstruct 3D points on surfaces that are not contained in the preoperative 3D model, resulting in a percentage of outliers prohibitively large for existing registration algorithms to work. To overcome this, the proposed system includes a deep learning-based model that automatically segments the arthroscopic images and identifies the regions of interest in which points will be reconstructed.

In summary, the paper presents the following key contributions: (1) A proof of concept of a low-cost SL system, comprising a calibrated laser projector and a standard arthroscope, that enables the detection of the projected light contour for inferring 3D point data intraoperatively and accomplishing the registration; (2) a deep learning-based model designed for automatic segmentation of the arthroscopic video that identifies the regions of the arthroscopic images that correspond to structures in the preoperative anatomical model, enhancing precision and efficiency in the registration process; and (3) a novel data augmentation technique that improves the performance of the automatic arthroscopic image segmentation model by synthesizing the laser projection in the arthroscopic images.

The experimental results validate the effectiveness of our approach in both phantom and real arthroscopic data, reinforcing its practical utility and potential impact in the field of surgical navigation for arthroscopy.

## Related work

SL is a method for 3D surface reconstruction based on triangulation. SL has been used in laparoscopic and knee arthroscopic surgery, mapping textureless organ surfaces. Hayashibe et al. [7] developed a two-endoscope system to perform 3D measurements and visualization. The two endoscopes were inserted into the abdominal cavity and an optical galvano scanner controlled a laser beam strip. Reiter et al. [17] also used a two-endoscope system, which included a projector and a dichroic beam splitter, enabling the projection of an invisible pattern to the surgeon. Schmalz et al. [19] developed a SL system based on a single-shot approach. Despite the endoscope’s small 3.6 mm diameter, the camera has a blind spot, resulting in the loss of information. Furthermore, the contrast of the color rings is relatively low, and the system has never been tested underwater. Long and Nagamune [10] introduced a system for arthroscopic knee procedures, comprising an arthroscope, an optical fiber for laser beam projection, and a prism. The common aspect to all these approaches is that they require the camera to be rigidly attached to the light projector, which may lead to issues associated with small baselines, such as increased errors in surface reconstruction.

Edgcumbe et al. [4] developed the Pico Lantern, a compact laser projector device designed for laparoscopic surgery. The Pico Lantern system comprises a projector and a fixed checkerboard for tracking purposes, allowing it to move freely relative to the laparoscope within the abdominal cavity. The downside is that the Pico Lantern device measures approximately 17 mm, being about 3$\times$ the size of a standard arthroscopic portal. Clancy et al. [3] presented a nonrigid SL system with a 1.7 mm diameter probe. Later, Lin et al. [9] proposed further developments in on-the-fly self-calibration to estimate the relative positions, enabling free-hand manipulation during surgery. However, the variations in tissue properties, scattering, and other factors introduced ambiguities in pattern decoding, and the resulting density of 3D points was very low [3, 9].

The proposed off-the-shelf laser scanner system differentiates itself from existing literature by offering a cost-effective arthroscopy solution. The proposed SL system has real-time extrinsic calibration, eliminating the need for rigid camera-projector attachment. Additionally, our system incorporates a simple line pattern, eliminating ambiguities in pattern decoding, simplifying the system design, and facilitating image projection detection and calibration.

## Methods

**Fig. 2:**
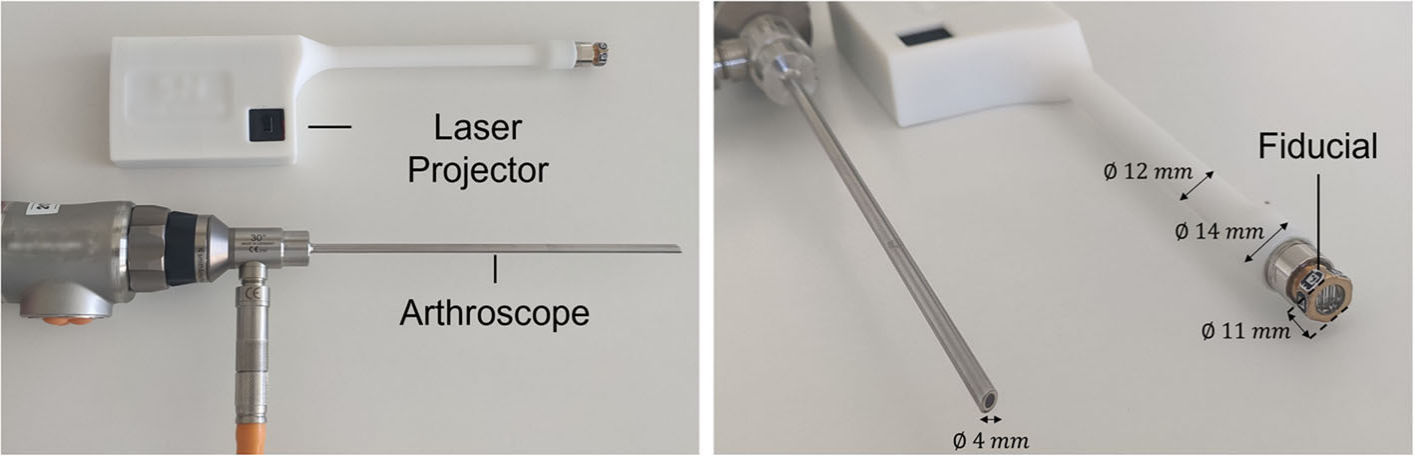
Proposed SL system setup with a standard arthroscope and laser projector instrumented with a fiducial. The relative pose between the arthroscope and the laser projector is known at every frame-time instant by tracking the laser fiducial

The SL system consists of a line-structured light projector and an arthroscope. The design is illustrated in Fig. 2. The laser projector is instrumented with a visual marker to enable tracking at every frame-time instant by the arthroscopic camera. Since this provides the extrinsic calibration between both SL system components, the arthroscope can go through one portal and the laser beam through the other, and both can move independently, being more versatile than existing methods.

In the current implementation of VBSN, the surgeon rigidly attaches a marker to bone (World Marker, WM) whose pose in camera coordinates can be determined at each time instant by tracking the visual markers [16]. This enables 3D points reconstructed in different frames to be represented in the same WM coordinate system that does not move with respect to the anatomy. As depicted in Fig. 1, the final step of registration aligns the 3D points reconstructed intraoperatively with a 3D model obtained preoperatively. Refer to Fig. 1 in the supplementary material for a schematic representation of the different coordinate systems and their relationships. Our pipeline for generating the 3D model consists of segmenting bone and cartilage structures from an MRI of the patient’s joint, applying the marching cubes algorithm [11], and smoothing the resulting 3D model.

This section provides a comprehensive overview of our laser scanner and presents an automatic segmentation model for identifying the regions of interest within arthroscopic images (femur bone and cartilage) such that 3D points reconstructed in other anatomical structures are not considered during registration. Lastly, the registration algorithm employed in this work is described.

### Laser scanner

The proposed SL system involves three main steps: detection of the laser contour in the arthroscopic image, calibration of the plane of light of the laser projector and 3D point reconstruction through triangulation. These steps are described below.

#### Detection of the laser contour

**Fig. 3:**
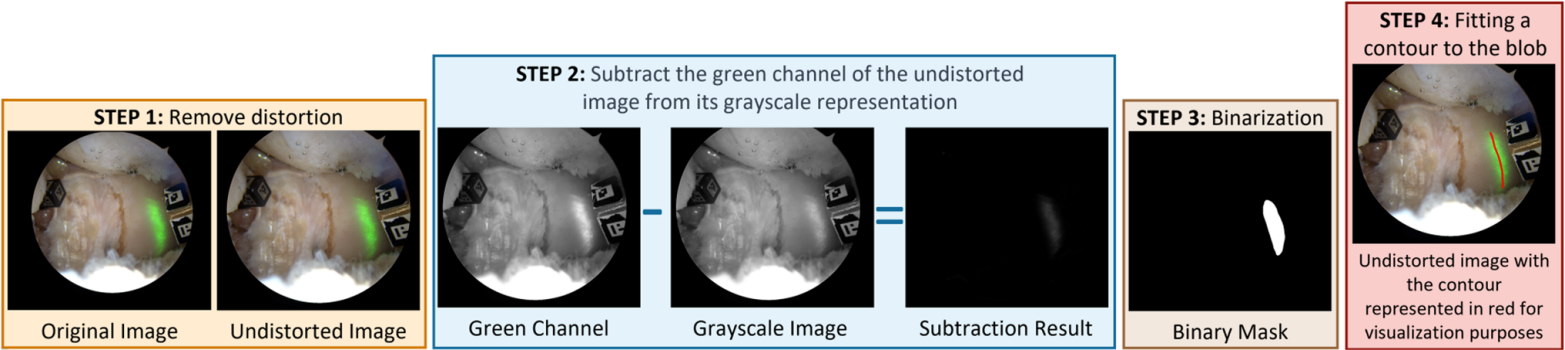
Laser detection pipeline: for every input frame, distortion is removed and the resulting undistorted image is both converted to grayscale and used for retrieving the green channel. The subtraction of these two images is binarized and blob detection is performed. The central line of laser projection is detected using a PCA-based approach, yielding the detection of the laser line

Fig. 3 depicts the main steps of the pipeline for detecting the laser projection. Firstly, the distortion of the input image is removed both for retrieving the green channel and the grayscale image. By subtracting these two images and binarizing the result, the obtained binary image provides the segmentation of the laser projection. Since the laser projection exhibits significant dispersion, it appears as blobs in the binary image. Blob detection is then performed and a PCA-based approach fits a contour to the blob by (i) extracting the direction of maximum variance, (ii) sampling the blob along that direction, and (iii) for each sample determining the midpoint of the line segment contained in the blob with perpendicular direction. The resulting contour is depicted in red in the final step of Fig. 3.

#### Projector calibration

Preoperatively, the laser projector must be calibrated, i.e., the equation of the plane of light in laser coordinates must be estimated. To accomplish this, a setup consisting of a planar target instrumented with visual fiducials is considered. By pointing the laser into the target, a line is projected and a calibration image showing the planar target, the laser projection and the laser marker simultaneously is acquired. Both the camera and the laser projector are moved to enable the acquisition of a calibration set with distinct poses. For each calibration image, the laser line projection is detected as described previously and 3D points are reconstructed in camera coordinates by intersecting backprojection rays with the planar target. These points are then transformed to laser coordinates using its tracked pose. After all calibration images are processed, a set of 3D lines is obtained, which is given as input to a RANSAC-based [5] plane fitting algorithm. Figure 2 in the supplementary material illustrates the calibration setup.

#### 3D point reconstruction

The basic principle of a SL system involves projecting a light pattern onto the scene and detecting it by a camera. The intersection of the light plane transformed to the camera coordinate system with the camera ray yields the 3D coordinates of the point (refer to Fig. 3 in supplementary material for a schematic representation). Performing this process for all points within the detected laser contour, we obtain a 3D contour in camera coordinates. Then, we can transform these points to the reference frame of the WM attached to the bone. By repeating this process for a set of frames, we achieve the reconstruction of a denser point cloud representing the anatomical structures. In Sect. 5.2, registration tests with data obtained by the proposed algorithm are performed.

### Arthroscopic video segmentation

**Fig. 4:**
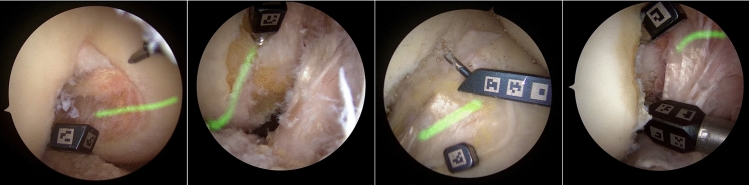
Example images of the synthetic laser projections. We propose a method that takes as input arthroscopic images without any laser projection and outputs the same image with a realistic projection of the laser. These generated images are used to train the semantic segmentation model

As previously discussed, the objective of the proposed pipeline is to register a preoperative femur bone and cartilage model with intraoperative data. However, due to the presence of other anatomical structures such as proximal tibia, the anterior and posterior cruciate ligaments and the meniscus, it is impossible to capture arthroscopic footage containing solely the structures of interest. These additional structures are not contained in the preoperative model, and, hence, should be removed from the reconstructed point set obtained with the structured light system. Therefore, we propose an automatic segmentation model designed for arthroscopic videos. The considered architecture is a standard U-Net [18] and is depicted in the supplementary material (Fig. 4). The loss function used is $1-\text {DICE}(T,P)$, where $\text {DICE}(T,P)$ is the DICE score [20] defined as

1 $$\begin{aligned} \text {DICE}(T,P)=\frac{2\sum _{i}^{N} T_i P_i}{\sum _{i}^{N}T_i + \sum _{i}^{N}P_i} \end{aligned}$$

where *T* is the ground-truth segmentation, *P* is the inferred segmentation, *i* is a pixel, and *N* is the number of pixels. The DICE score is one when the inferred and ground-truth segmentations overlap perfectly, and zero when there is no overlap.

All input images are normalized using the contrast limited adaptive histogram equalization, as outlined in [14], to enhance image contrast. Furthermore, standard data augmentation, including image rotation, translation, scaling, and flipping, is applied. This step is important because the dataset is limited in size and data augmentation is an effective strategy for artificially increasing the amount of training samples [13].

Two segmentation models, the no laser augmentation (NLA) model and the laser augmentation (LA) model, were trained following identical procedures except for the training datasets. The NLA model was trained solely using the dataset described in Sect. 4.1.1, while the LA model was trained by also considering images with synthetic laser projection. This novel data augmentation technique will be described below.

#### Data augmentation using synthetic laser projection

For each image in the training dataset, a new image containing synthetic laser projection is generated as follows. Considering the corresponding ground-truth binary mask, a random pixel within the region of interest is initially selected. Using the registration and the camera pose relative to the image, the forward projection ray that goes through the pixel is intersected with the 3D model, yielding a 3D point. Then, a random 3D vector is chosen, that, together with the 3D point, define a 3D plane. This 3D plane corresponds to the synthetic laser plane of light and is afterward intersected with the 3D model, yielding a 3D contour. By backprojecting this contour onto the image, a synthetic laser projection is generated. Finally, the laser light dispersion is simulated by applying a Gaussian intensity distribution centered at the backprojected 2D contour. Figure 4 depicts different synthetic laser projections obtained with the described approach.

### Registration

Following the acquisition of arthroscopic footage with laser projection using the pipeline detailed in Sect. 3.1, and filtering outlier 3D points with one of the segmentation models from Sect. 3.2, we obtain a dense point cloud representing the anatomical structures. The next step involves accomplishing registration using the method presented in [15] for curve-surface registration. It is a method for global registration that automatically finds pairs of matching points in the curve and in the surface, along with their tangents and normals, for estimating the rigid transformation between the preoperative model and the patient’s anatomy. For each pair of points from the curve with associated tangents, the algorithm finds all matching pairs of points and associated normals on the surface using a set of conditions that depend on the differential information (tangents and normals). Then, an hypothesize-and-test framework finds the rigid transformation that best aligns the reconstructed curve with the preoperative surface. A final standard ICP step [2] is performed for refining the solution.

## Experiments

This section details the datasets, experimental setups, and evaluation metrics used to assess the performance of the segmentation models (mentioned in Sect. 3.2) and registration algorithm (described in Sect. 3.3) using our laser scanner.

### Arthroscopic video segmentation

#### Dataset description

The dataset employed in the development and evaluation of the deep learning model comprises arthroscopic images obtained from five distinct cadaver specimens, of which two contain sequences showing laser projections. In order to use these two specimens solely for testing and following common practice in deep learning, where it is advisable to have distinct training and testing subjects, the data from the remaining three cadavers was exclusively allocated for training. The dataset images were selected by (i) sampling each arthroscopic video at a frame rate of 0.3 frames per second, (ii) since the camera pose at each frame is known, clustering poses using a threshold of 1.5 mm and $20^\circ$, and (iii) randomly selecting one image from each cluster. This selection scheme resulted in a total of 260 training images approximately evenly distributed among the cadavers and 58 testing images divided into 35 images without laser projection and 23 images with laser projection. The ground-truth binary masks (labels corresponding to the region of interest and background) for each image were obtained through manual annotation by experienced engineers.

It is important to emphasize that the arthroscopic images of the training dataset do not contain any structured light projection. In order to generalize the model to handle images with structured light projections, we use the novel data augmentation strategy for generating synthetic laser projections described in Sect. 3.2.1.

#### Training details

The models were trained on four NVIDIA GeForce GTX 1080 Ti 12GB, resulting in a training time of approximately in 2-3 h. Regarding the training hyper-parameters, a learning rate of 1e-4 and batch size of 16 were employed. The optimization process utilized the AdamW optimizer [12]. Furthermore, the learning rate was reduced when the validation loss reached a plateau, and an early stopping mechanism was implemented with a patience of 50 epochs to minimize training time.

#### Evaluation metric

The metric used to evaluate the performance of the automatic segmentation models in the test set is the DICE score (Eq. 1).

### Registration

#### Dataset description

**Fig. 5:**
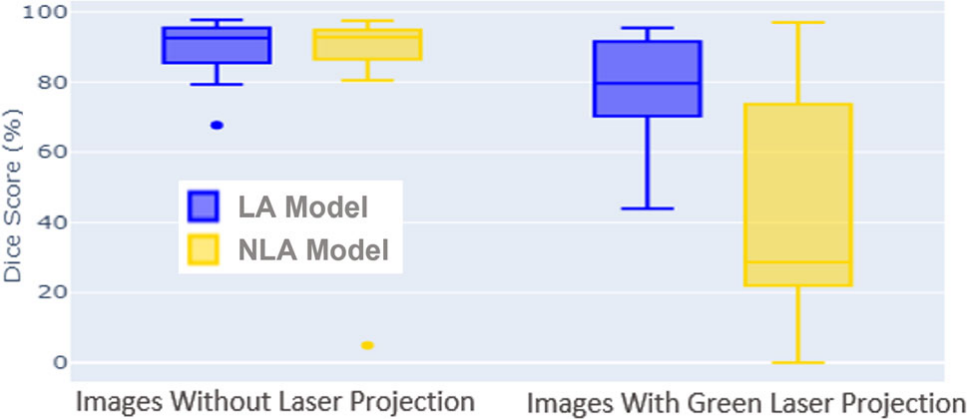
Performance of the segmentation models trained with (blue) and without (yellow) laser augmentation (LA) on the test set images. Both models perform comparably when testing on images without laser projection. For images containing laser projection, only the model trained with LA performs well

Considering the anatomies of interest of this work, we prepared a hip and a knee phantom (refer to Fig. 5 and 6 in the supplementary material) for testing. We acquired 6 laser scanning sequences of the bone surface for about 30 s and then performed registration. A video provided in the supplementary material illustrates this process for the case of the hip model. Furthermore, an experiment using an ex-vivo knee was conducted by following a similar strategy as the one in dry model in terms of data acquisition. Registration was then performed both by considering all the reconstructed points and by previously segmenting the arthroscopic images using the models described in Sect. 3.2, i.e., the NLA model and the LA model.

#### Experimental setup

All experiments were performed in a PC that is connected in-between camera tower and display. The PC is equipped with a frame grabber Datapath Limited DGC167 in an Intel Core i7 4790 and a GPU NVIDIA GeForce GTX950 that was able to run the pipeline in HD format at 60fps with latency of 3 frames.

#### Evaluation metric

We follow the evaluation protocol used in [16] and consider a set of 4 control points (refer to Fig. 7) for measuring the quality of registration with the proposed laser scanner. For all registration solutions, the control points are represented in WM coordinates and a centroid corresponding to each control point is determined. The RMS value of the distance between each transformed point and the corresponding centroid provides a metric for the precision of registration.

## Results and discussion

This section reports and discusses the results obtained in the experiments described in Sect. 4. Additionally, registration results obtained with the proposed method are compared with the existing touch probe approach.

### Arthroscopic video segmentation

**Fig. 6:**
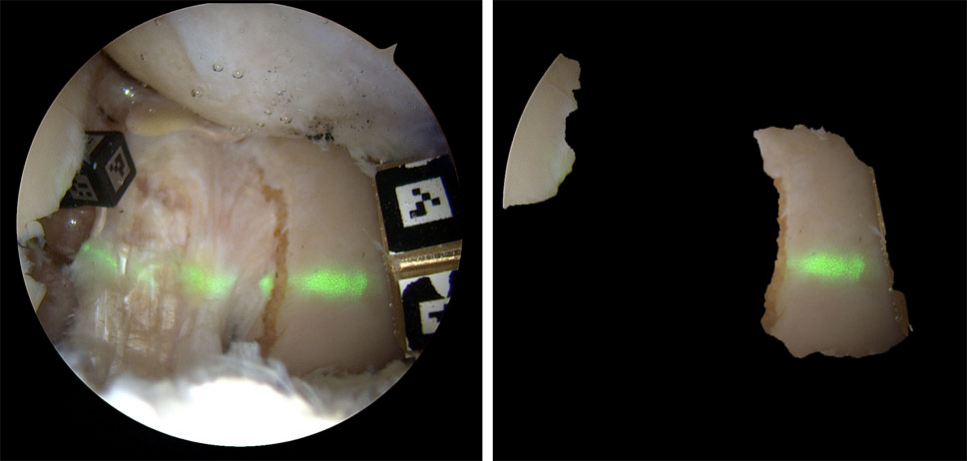
(left) Arthroscopic frame with laser projection. (right) Segmentation result where only bone and cartilage structures are considered. Pixels with laser projection corresponding to nonrigid structures (e.g., ligaments inside the intercondylar region) are removed

Fig. 5 shows quantitative results of both automatic segmentation models using the DICE score (Eq. 1). For a qualitative evaluation, refer to Fig. 7 in the supplementary material. As previously mentioned, the test set comprises two cadavers, each featuring images with and without laser projection. For arthroscopic images without laser projection, the automatic segmentation models demonstrate good and comparable performance, achieving a DICE score of $0.86\pm {}0.20$. However, in cases with laser projection, the NLA model in Fig. 5 does not perform well, having a DICE score of $0.42\pm {}0.32$. This is expected because this model does not have target domain images in its training dataset. The LA model (blue in Fig. 5), which incorporates the novel data augmentation technique described in Sect. 3.2.1, outperforms the NLA model for images with laser projection. The resulting performance metric indicates a DICE score of $0.80\pm {}0.13$. Figure 6 demonstrates the good performance of the LA model in a real arthroscopic image with laser projection. It is worth emphasizing that our training and testing datasets are notably smaller compared to standard computer vision datasets, e.g., [6]. Nevertheless, they were enough to show that the automatic segmentation of the structures of interest is accurate even when presented with images containing laser projections that were not part of the training dataset.

### Registration

This section reports experiments that assess the performance of the registration of a preoperative model with the patient’s anatomy. Tests in laboratory and using ex-vivo data are performed. The supplementary material includes two videos showcasing the registration process and its outcomes using the hip phantom and the ex-vivo specimen.

#### Experiments on dry model

**Fig. 7:**
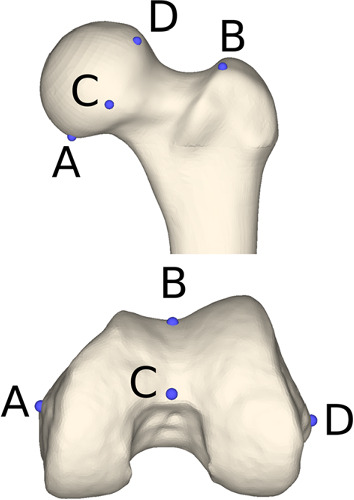
Control points (CP) for registration assessment on hip (top) and knee (bottom)

Considering the control points depicted in Fig. 7, the obtained registration errors are shown in Table 1 and demonstrate that high precision was achieved. In particular, errors in control point (CP) **C**, which corresponds to the region of acquisition of the laser scans, are below 0.5 mm. Also, for the remaining control points, all errors are below 1 mm, even in regions that are more than 5 cm away from the reconstructed area.

#### Ex-vivo experiments

**Fig. 8:**
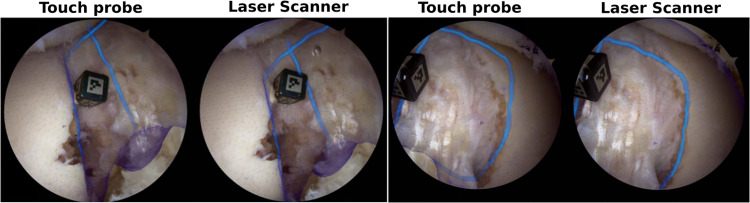
Qualitative assessment of the registration accuracy using augmented reality: the preoperative model (mesh grid) augmented with blue contours representing the intercondylar arc and Blumensaat’s line is overlaid with the arthroscopic images using the registration solutions obtained with the existing touch probe method and the proposed laser scanner approach. Both methods performed well, as can be seen by the good alignment of the model with the anatomy in two distinct arthroscopic views

Results shown in Table 1 evince the need for the segmentation model. Without segmentation (No Segm), the reconstruction is highly outlier-contaminated, precluding the registration algorithm from performing well. When segmentation is done by the NLA model, results are poor due to oversegmentation of the arthroscopic images, which cause not only outliers but also model regions to be removed, resulting in a very sparse reconstruction. Results obtained with the LA model are comparable with the ones reported in [16], with errors below 2.2 mm for all control points and, in particular, below 1 mm in CP **C**, which corresponds to the acquisition region (intercondylar region). For this particular knee, registration with a touch probe was performed 7 times and the results provided in Table 1 show that the error in CP **C** is identical to the one obtained with LA. Overall, only a slight improvement in performance is observed (on average, an error 0.45 mm lower), demonstrating that both methods are comparable in terms of accuracy. Figure 8 shows the mesh of the preoperative model overlaid in the anatomy using augmented reality for both the registration obtained with a touch probe [16] (left) and with our laser scanner (right). It can be seen that, although the registration solutions are not identical, they are similar and it is difficult to decide which one is the most accurate. This further confirms that our approach is capable of performing intraoperative registration with satisfactory quality.

**Table 1:** Registration precision (mm) in phantom and ex-vivo. For the ex-vivo case, the reconstructed points are fed to the registration algorithm in three different manners: (i) without considering the segmentation model (No Segm) and by segmenting the arthroscopic video with (ii) the model with no laser augmentation (NLA), and (iii) the model with laser augmentation (LA). Errors obtained by feeding points reconstructed with a probe are given in the rightmost column

	Phantom		Ex-vivo Knee			
CP	Hip	Knee	No Segm	NLA	LA	Probe
A	0.67	0.54	19.40	28.81	2.12	1.52
B	0.92	0.71	29.39	24.17	1.95	1.29
C	0.44	0.36	11.77	9.76	0.76	0.89
D	0.42	0.88	46.22	29.76	1.86	1.19

## Conclusion

We presented a proof-of-concept SL system designed for arthroscopy, combining the arthroscopic camera with a low-cost laser projector. The prototype’s design is compact enough to fit inside the arthroscopic portals, allowing for real-time computation of 3D point data during surgical procedures. To increase the system robustness, a deep learning-based model was introduced to segmenting areas within the arthroscopic video that belong to surfaces in the preoperative 3D model. By reconstructing the laser projection within these regions, it is possible to recover a 3D point set that can be registered with the preoperative model. The experimental section shows promising results, proving that it is possible to register the intraoperative arthroscopic environment with a preoperative model accurately without the need for physical digitization of anatomical surfaces. Nevertheless, the presented system still has some limitations, with the major one being the large diameter of the laser projector that (i) is greater than the size of standard arthroscopic portals (6 mm) and (ii) hinders the simultaneous visualization of the WM, the laser fiducial and the laser projection by the arthroscopic camera. Additionally, the laser beam would benefit from better collimation. As future work, we intend to decrease the diameter of the projector to a maximum of 6 mm and plan to improve collimation with better hardware. This innovative concept paves the way for touchless registration in arthroscopic procedures, offering potential solutions for complex scenarios such as those encountered in procedures like FAI or shoulder surgeries, where minimal invasive vision-based navigation systems are not yet available.

### Supplementary Information

Below is the link to the electronic supplementary material.

**Supplementary information:** In addition to the main text of this article, we have included supplementary information as an attachment. (pdf 459KB)

**Supplementary information:** In addition to the main text of this article, we have included supplementary information as an attachment. (mp4. 69,155KB)

**Supplementary information:** In addition to the main text of this article, we have included supplementary information as an attachment. (mp4. 80,076KB)
