# Supplementary material for: Structured light for touchless 3D registration in video-based surgical navigation
Source: Int J Comput Assist Radiol Surg. 2024 May 30;19(7):1429–37. doi: 10.1007/s11548-024-03180-5 (PMC11230986; doi:10.1007/s11548-024-03180-5)
Supplement: Supplementary file 1 — Supplementary information: In addition to the main text of this article, we have included supplementary information as an attachment. (pdf 459KB) [file 11548_2024_3180_MOESM1_ESM.pdf]

Supplementary Material

Structured Light for Touchless 3D Registration in Video-based Surgical Navigation

Tânia Baptista, Miguel Marques, Carolina Raposo,

Luís Ribeiro, Michel Antunes, João Barreto

This document provides information useful for better understanding the methods and concepts described in the main text. Additionally, two videos are also provided:

- video1.mp4: a comparison of the registration process with the touch probe and with the proposed system using the hip phantom.
- video2.mp4: registration with the proposed system in an ex-vivo specimen.

3 Methods

Fig. 1 provides a schematic representation of the complete pipeline of touchless 3D reconstruction and registration. For each frame containing a laser projection, the tracked laser pose T_L is used to transform the calibrated plane of light onto the reference frame of the camera. Simultaneously, the laser projection is detected in the image and the 3D triangulation process described in Fig. 3 is applied for reconstructing 3D points. These points are represented in the reference frame of the world marker (WM) using its tracked pose T_W. Registration with a pre-operative model is afterwards determined, yielding rigid transformation T_R.

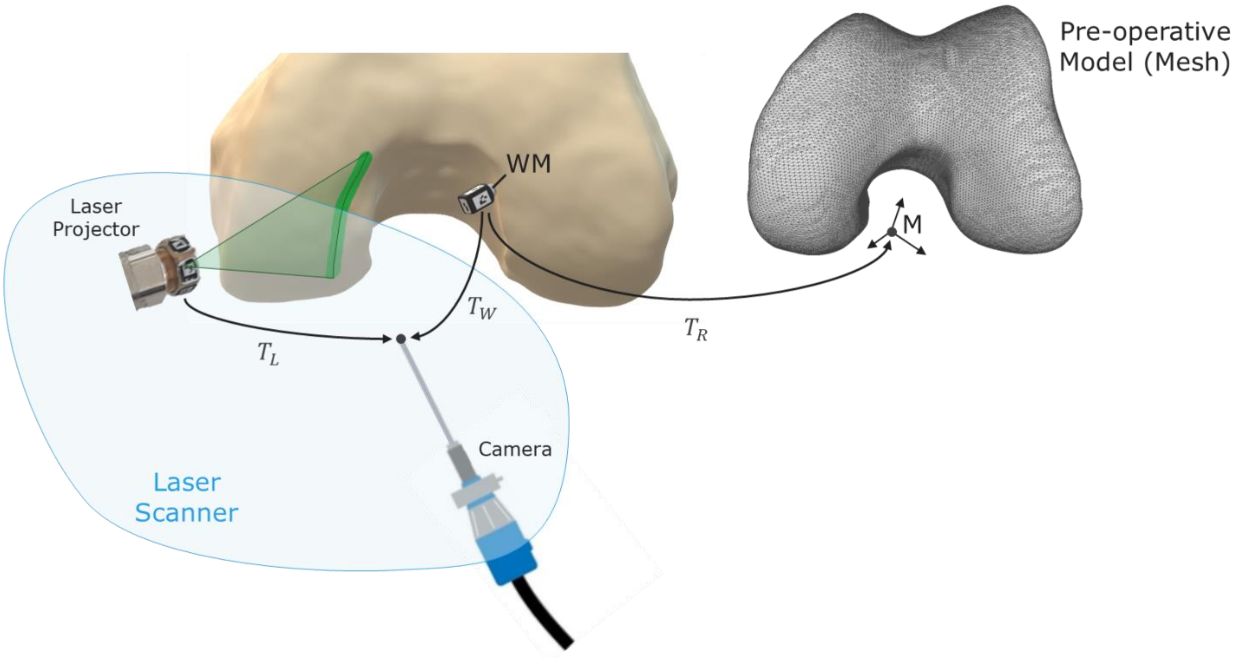

Fig. 1 Schematic representation of the complete pipeline. For each incoming frame, the plane of light of the laser projector is transformed to the camera reference frame using the laser's pose T_L. After the projected laser contour is reconstructed, it can be represented in world marker coordinates using transformation T_W. Registration between the reconstructed contours in WM coordinates and the coordinate system M of the pre-operative model is represented by the rigid transformation T_R

3.1 Laser Scanner

The reference frame of the laser projector is given by the visual marker that is rigidly attached to it and can be seen in Fig. 2. Pre-operatively, the equation of the plane of light of the laser projector must be estimated in this reference frame such that, intra-operatively, by tracking the laser's visual marker, it can be transferred to the reference frame of the camera for each incoming frame and triangulation can be performed (refer to Fig. 3). Registration is then accomplished by aligning the triangulated points and the pre-op model (Fig. 1).

Determining this plane equation is referred to as *projector calibration*. This is performed by acquiring a set of images as depicted in Fig. 2. For each image, the camera tracks the markers in the planar target for determining its plane pose. The laser projection is detected, and 3D points are reconstructed by intersecting back-projection rays with the target plane. These 3D points are then transferred to the laser's reference frame by tracking its visual marker with the camera. Repeating this procedure for multiple frames yields a set of 3D lines that are fitted to a 3D plane (the plane

of light of the laser projector) using a robust plane-fitting approach. Fig. 2 also shows the calibration error as the distance between the 3D points of the laser projection, computed as described above, and the intersection line between the planar target and the laser light plane. The errors are calculated using frames not included in the calibration set. A median error of 0.15mm was obtained, which demonstrates that the plane of light is accurately calibrated.

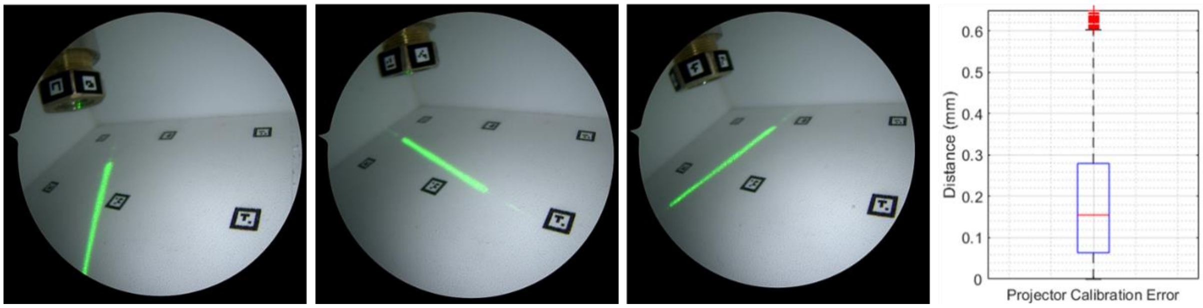

Fig. 2 Example images of the dataset acquired for calibrating the plane of the light of the laser projector. The calibration setup consists of a planar target with printed visual markers placed in the bottom of box that is filled with water. The laser projector is inserted into the box and images showing simultaneously the projected line, the laser fiducial and planar target are acquired with the arthroscope. Both the arthroscope and the laser projector can move freely during image acquisition. The calibration set contains 30 images. To evaluate the calibration of the projector, an additional set of 30 images with light planes in different positions than the calibration set is acquired. Once the pose of the planar target is determined, the resulting line of intersection between planar target and projector plane is calculated. The laser projection is detected, and 3D points are reconstructed. The projector calibration error is defined as the distance between the resulting line of intersection of the two planes and the reconstructed 3D laser points

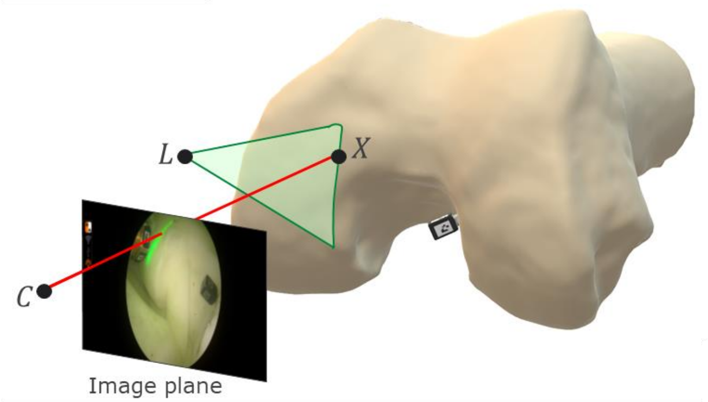

Fig. 3 Optical triangulation principle. The laser projector emits a light plane L (shown in green) that intersects the scene in a 3D curve. This curve is imaged by the arthroscopic camera, as shown by the bright green 2D contour on the image plane. For each pixel of the 2D contour, a back-

projection ray (red line) that goes through it and the camera's optical center C is created. By intersecting the ray with the light plane, the 3D point X is reconstructed

3.2 Arthroscopic Video Segmentation

The automatic segmentation model used is a standard U-Net.

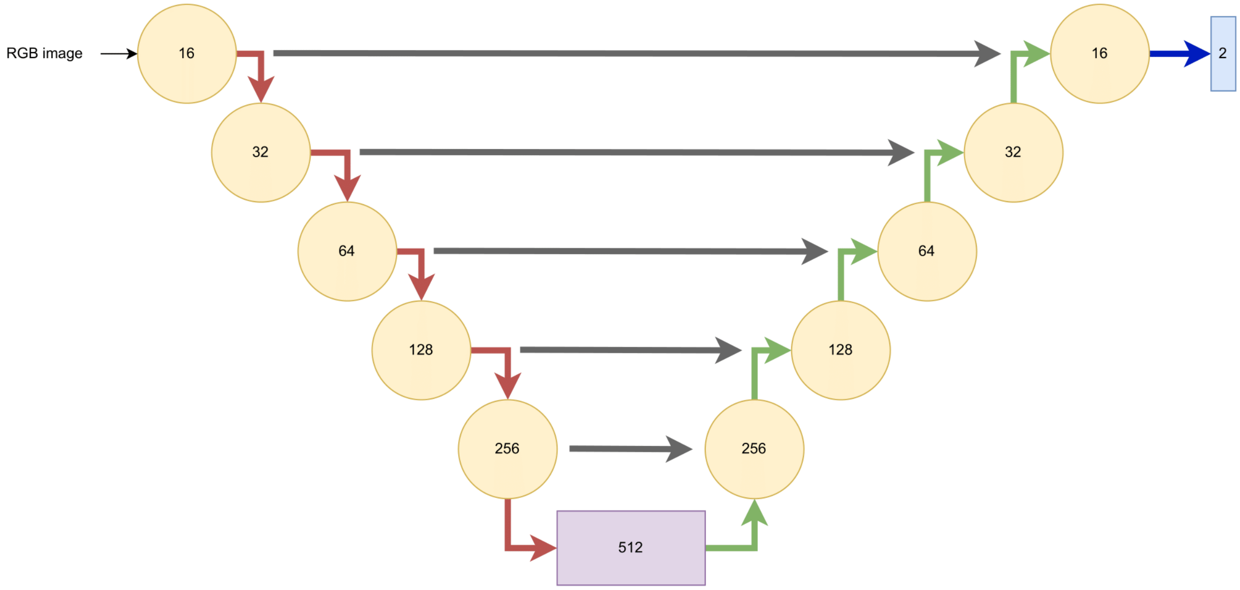

Fig. 4 The segmentation model is a U-Net architecture with depth 5. Each circle represents two 2D convolutions, with the number in the circle representing the number of output channels. Red arrows indicate max pools, green arrows indicate up 2D convolutions and grey arrows are skip connections. The purple block is the network's bottleneck. The last blue block is only one 2D convolution with kernel size of one. Besides the last block, all 2D convolutions have a kernel size of three and are followed by a batch normalization and ReLU activation

4 Experiments

4.2 Registration

This section describes the experimental setups used in laboratory tests performed on knee and hip dry models. The phantoms were designed to simulate the configuration of real arthroscopic procedures.

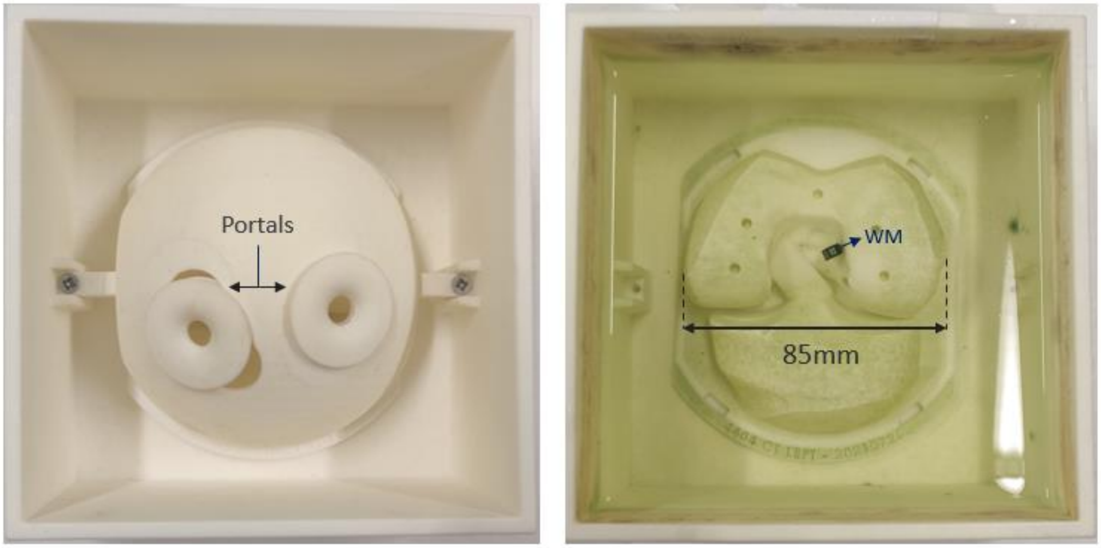

Fig. 5 Dry knee model setup. A phantom containing 3D printed models of a distal femur with the posterior cruciate ligament and a proximal tibia in a 90º flexion configuration was used in the experiments. This phantom is attached to a box that is filled with water and contains a dome with two openings that simulate the arthroscopic portals typically opened when performing an ACL reconstruction surgery. A visual marker for tracking the arthroscopic camera was rigidly attached to the femur model. During the experiments, the arthroscopic camera is inserted into one portal and the laser projector into the other

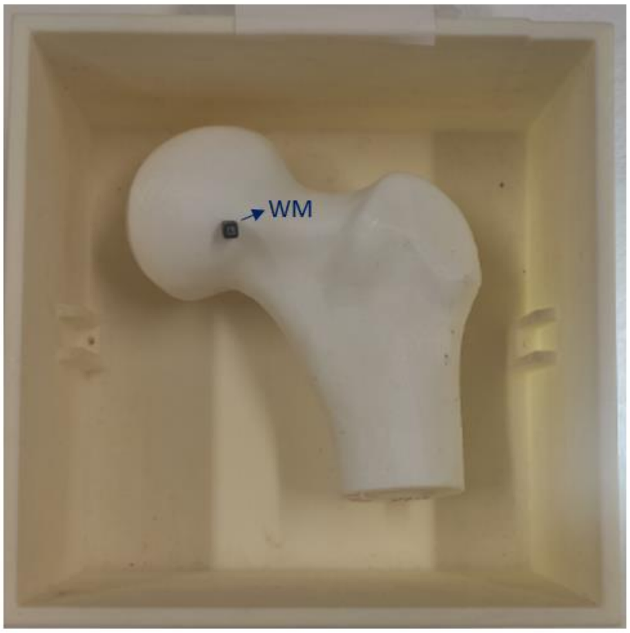

Fig. 6 Dry hip model setup. A phantom containing a printed 3D model of a proximal femur with a small cam deformity is considered. The visual marker is rigidly attached to the model near the region of deformation that is accessible during arthroscopic surgery. The model is attached to a box filled with water

5 Results and Discussion

5.1 Arthroscopic Video Segmentation

The introduction of the novel method for generating synthetic images with laser projection substantially improves the performance of the model for images presenting laser projection. Fig. 7 compares both segmentation models (trained without and with images having laser projection, i.e., NLA and LA model, respectively) on two test images with and without laser projection.

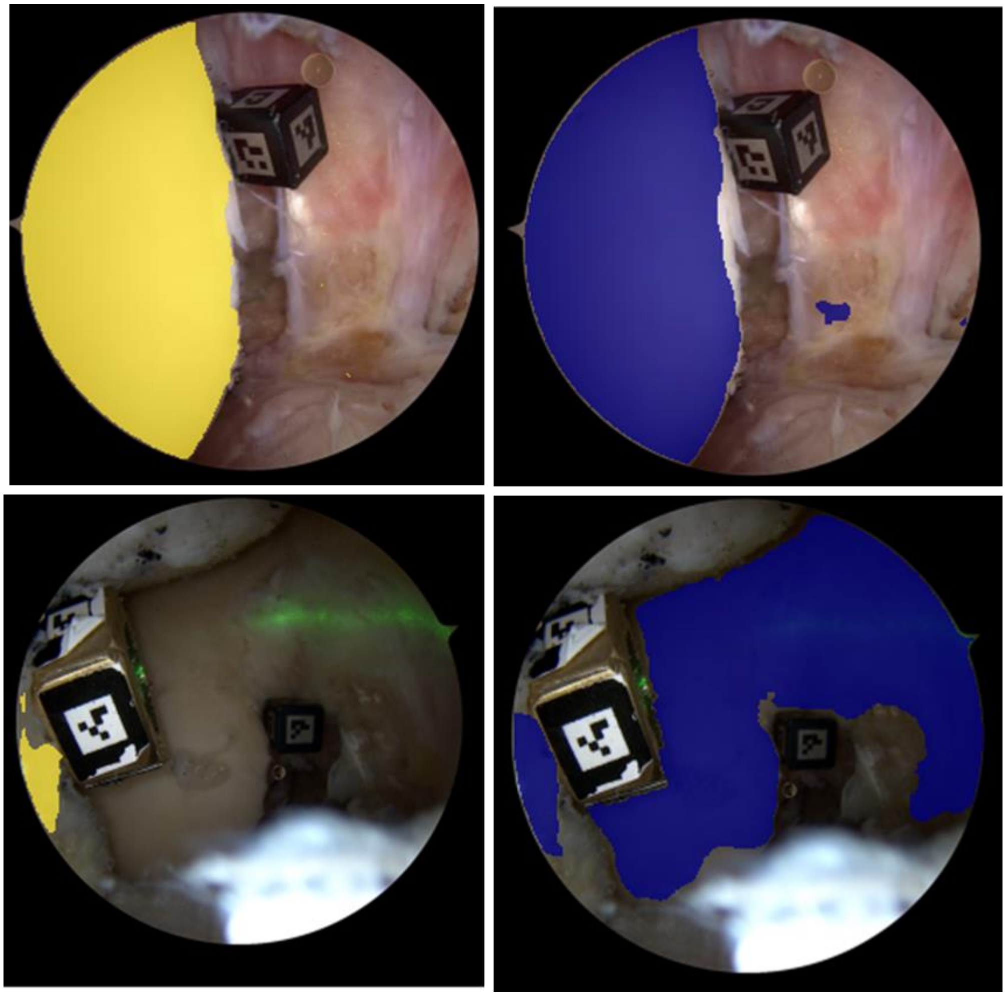

Fig. 7 Qualitative evaluation of the segmentation results for two arthroscopic frames (rows). The left column (yellow) shows the result of the automatic segmentation model trained without images having laser projection (NLA model). The right column (blue) shows the result of the automatic segmentation model trained with laser projection augmentation (LA model). When feeding the NLA model with images having laser projection, it performs poorly and fails to segment most of the region of interest. On the other hand, the LA model performs well both on images without (top row) and with (bottom row) laser projection
